# Association of immune checkpoint inhibitors with SARS-CoV-2 infection rate and prognosis in patients with solid tumors: a systematic review and meta-analysis

**DOI:** 10.3389/fimmu.2024.1259112

**Published:** 2024-06-03

**Authors:** Lin Sun, Fangmin Zhao, Yuying Xiang, Shuyi Chen, Qijin Shu

**Affiliations:** ^1^ The First School of Clinical Medicine, Zhejiang Chinese Medical University, Hangzhou, Zhejiang, China; ^2^ Department of Oncology, The First Affiliated Hospital of Zhejiang Chinese Medical University (Zhejiang Provincial Hospital of Traditional Chinese Medicine), Hangzhou, Zhejiang, China

**Keywords:** SARS-CoV-2, immune checkpoint inhibitors, cancer, infection, mortality, meta-analysis

## Abstract

The rate and prognosis of severe acute respiratory syndrome coronavirus 2 (SARS-CoV-2) infection in patients with solid cancer tumors actively treated with immune checkpoint inhibitors (ICIs) have not been fully determined. The goal of this meta-analysis was to explore this issue, which can be helpful to clinicians in their decision-making concerning patient treatment. We conducted a thorough search for relevant cohort studies in the databases PubMed, Embase, Cochrane Library, and Web of Science. Mortality and infection rate were the primary endpoints, and the incidence of severe or critical disease was the secondary result. A total of 6,267 cases (individual patients) were represented in 15 studies. Prior exposure to ICIs was not correlated with an elevated risk of SARS-CoV-2 infection (relative risk (RR) 1.04, 95% CI 0.57–1.88, z = 0.12, *P* = 0.905) or mortality (RR 1.22, 95% CI 0.99–1.50, z = 1.90, *P* = 0.057). However, the results of the meta-analysis revealed that taking ICIs before SARS-CoV-2 diagnosis increased the chance of developing severe or critical disease (RR 1.51, 95% CI 1.09–2.10, z = 2.46, *P* = 0.014). No significant inter-study heterogeneity was observed. The infection and mortality rates of SARS-CoV-2 in patients with solid tumors who previously received ICIs or other antitumor therapies did not differ significantly. However, secondary outcomes showed that ICIs treatment before the diagnosis of SARS-CoV-2 infection was significantly associated with the probability of severe or critical illness.

**Systematic review registration:**

https://www.crd.york.ac.uk/prospero/#recordDetails PROSPERO, identifier CRD42023393511.

## 1 Introduction

In late 2019 and early 2020 the outbreak of severe acute respiratory syndrome coronavirus 2 (SARS-CoV-2) rapidly spread to become a global pandemic and a serious public health emergency, and the associated syndrome was named coronavirus disease 2019 (COVID-19) (1). According to reports, patients with cancer are more likely to develop SARS-CoV-2 infection than healthy individuals, and this patient population is characterized by rapid disease progression, a higher proportion of individuals needing more intensive care, and a higher fatality rate (2–4). Therefore, there was a general trend at the beginning of the pandemic to stop cancer treatment because it was not known whether such treatments would affect the infection rate and prognosis of SARS-CoV-2. One of the most important cancer treatments in oncology is the use of immune checkpoint inhibitors (ICIs), a type of immunotherapy that improves patient outcomes in cancer cases. ICIs have shown significant efficacy in various cancers, such as lung cancer, melanoma, head and neck squamous cell carcinoma, and classical Hodgkin’s lymphoma (5). The approved ICIs are monoclonal antibodies that block the proteins CTLA-4, PD-1, or PD-L1.

Due to tumor depletion, malnutrition, and decreased immune function caused by anti-tumor therapy, patients with malignant tumors are susceptible to SARS-CoV-2 infection. Therefore, most guidelines on the effects of ICIs on the course of COVID-19 have focused on their potentially harmful effects, especially on the development of the most serious inflammatory complications. Moreover, it is generally recommended to delay the use of ICIs in the SARS-CoV-2 environment. The National Comprehensive Cancer Network (NCCN) advises delaying antitumor treatment for patients who test positive for SARS-CoV-2; the precise amount of time depends on the severity of the infection (6), which can be a useful indicator in the absence of other factors. Other organizations have issued recommendations for treating patients with cancer. The German Society for Hematology and Medical Oncology issued a guideline that advocated delaying or stopping antineoplastic medicines during the current pandemic (7). The National Institute for Health and Care Excellence (NICE) advises that systemic anticancer treatment should ideally be delayed until the patient has been without severe symptoms for at least 10 days (8).

Because the majority of current guidelines recommend delaying ICI treatment, oncologists are becoming increasingly concerned about the safety of this treatment. Consequently, patients may have had their treatments postponed or permanently stopped, which could have had a significant impact on their prognosis (9–11). However, we note that there are many published studies in which the authors propose that ICI administration to patients with SARS-CoV-2 infection may be beneficial. According to Yekeduz et al. (12), the use of ICIs in patients with cancer during the pandemic proved safe. As reported by Pezeshki et al. (13), ICIs can be used as immunotherapy against SARS-CoV-2 in patients without cancer because they increase T-cell proliferation and activation. Moreover, ICIs stimulate T cells and may suppress viral infection (14, 15). ICIs have been reported to increase the absolute lymphocyte count in patients with cancer, which is considered a strong prognostic signal and a marker of therapy response (16).

We reviewed prior research, including the molecular processes of ICIs and the associated pathophysiological mechanisms of SARS-CoV-2 infection, to investigate the causes. Through the mediation of cellular immunological and cytotoxic capabilities, CD8+ T cells play a critical role in regulating pathogen infection (17) and are involved in viral clearance (18). The number of T lymphocytes has been shown to continuously decline in the peripheral blood of individuals with SARS-CoV-2-induced diseases (19). This decrease was more pronounced in CD8+ T cells after analyzing the cell counts and traits of various T cell subsets (20), and the low proportion of CD8+ T cells was associated with the occurrence of acute respiratory distress syndrome (ARDS) in patients with SARS-CoV-2 activity (21). However, during the development of the disease, a vicious circle is formed due to the presence of exhausted CD8+ T lymphocytes that frequently overexpress the inhibitory receptors CTLA4 and PD-1/PD-L1, resulting in impaired effector function and low proliferative potential (22).

Malignant tumors often express the PD-L1 and/or PD-L2 ligands, which bind to PD-1 on T cells and cause T cell “exhaustion.” Thus, malignant cells can escape the anti-tumor defenses of the host. Through a variety of intricate activation mechanisms, ICIs cause T cells to activate, which kills cancer cells. The process begins with antigen presentation on the surface of antigen-presenting cells (APC) through the T cell receptor (TCR) and major histocompatibility complex (MHC) (23), and the costimulatory signal is controlled by the binding of B7–1 (CD80) or B7–2 (CD86) on the surface of APC to CD28 on the surface of T cells (24). Inhibitory T-cell receptors and their ligands include LAG3-MHC, CTLA4-B7, and PD1-PDL1. To restore the T-cell-mediated antitumor immune response, anti-PD-1/PD-L1 antibodies act on the effector phase of the cancer immune cycle and block the PD-1/PD-L1 pathway (25). By inhibiting CTLA-4 from attaching to B7, the anti-CTLA-4 antibody improves the ability of CD28s to bind to B7, activates T cells, and has antitumor effects (26, 27). These results indicate that ICIs may reduce the infection rate or poor prognosis of SARS-CoV-2 by increasing the T-cell response to viruses (28).

Based on the clinical study results and the deduced mechanisms mentioned above, we propose that ICIs are safe and will not affect the SARS-CoV-2 infection rate or prognosis of patients with solid tumors. However, only a few meta-analyses have summarized the safety of ICIs administered to patients with cancer in the context of SARS-CoV-2 (29, 30), and none of them are specific to patients with solid tumors. Therefore, to explore the safety of ICIs treatment and provide evidence-based treatment insights and risk recommendations for the potential interaction between ICIs and COVID-19, we conducted this meta-analysis to compare the SARS-CoV-2 infection rate and prognosis in patients with solid tumors receiving ICIs (compared with those patients who did not receive ICIs treatment). we screen qualified studies with unified and scientific evaluation criteria, and apply statistical methods to evaluate the infection rate, mortality and severity of patients with solid tumor.

## 2 Material and methods

### 2.1 Search strategy

The Preferred Reporting Items for Systematic Reviews and Meta-analyses (PRISMA) guidelines (31) were followed in the design and reporting of our study, which has been registered in the PROSPERO database (CRD42023393511). A thorough search plan was then developed. Studies published from December 1, 2020, to January 29, 2023, were retrieved from the following databases: PubMed, Embase, the Cochrane Library, and Web of Science. The following keywords were included in our search, based on the PICOS principle: “Immune Checkpoint Inhibitor,” “SARS-CoV-2,” “Neoplasms,” etc. The detailed search formulas are provided in **Supplementary Material 1**.

### 2.2 Criteria for inclusion and exclusion

The criteria for selecting studies were: (a) patients with solid cancer who received ICIs treatment were included in the study, (b) patients in the control group who received other types of antitumor therapies as comparators, and (c) SARS-CoV-2 infection and mortality were the primary outcomes, and severe/critical COVID-19 was the secondary outcome, and (d) cohort studies that computed RRs using pertinent statistics. In terms of the designation “severe” or “critical” illness, this was determined based on WHO guidelines (32, 33). We evaluated the criteria for severe respiratory disease, characterized by the need for an intensive care unit (ICU), or respiratory failure/acute respiratory distress syndrome (ARDS).

The following exclusion criteria were applied: (a) studies published only in abstract form, (b) studies in which patient data were missing for a group of patients, (c) studies involving patients with hematological malignancies, (d) studies in which data for patients receiving ICIs could not be distinguished from the data for the entire patient group, and (e) basic research, reviews, conferences, guidelines, editorials, comments, case reports, study protocols, and repeated publications.

### 2.3 Study selection

The screening procedure was performed by two independent authors who were blinded to one another. Full-text screening was performed to identify studies that met the inclusion and exclusion criteria. Disagreements between the reviewers were resolved through conversations with a third author.

### 2.4 Data extraction

One author extracted the data and created a specific table to house all study data, and another author double-checked the data extraction to ensure data accuracy, and the following details were recorded: name of the first author, publication year, country, study type, total sample size, proportion of males, median or mean age, tumor type, SARS-CoV-2 diagnostic method, ICIs interval before diagnosis of the virus, and study outcomes. The main outcomes were RRs with 95% CIs for infection rate and mortality, and the incidence of severe/critical disease was recorded as the secondary outcome. By extracting the total number of studies from the original information and the number of occurrences in the experimental and control groups, we calculated the incidence of events in each group and obtained the relative risk (RR) values. Specific information was obtained from published data or calculated using the original chart data.

### 2.5 Quality assessment

The Newcastle-Ottawa Scale (NOS) was used to rate the quality of the observational studies. Selection (4 points), comparability (2 points), and outcome (3 points) were the three elements of the scale (34, 35). If a study received at least seven out of nine overall responses, it was considered to be of excellent quality. Two reviewers evaluated the quality of the studies. A consensus was reached to address any discrepancies. The results of the quality assessment of the articles are summarized in **Supplementary Material 2**. The NOS ratings of the listed articles varied from 7 to 9, all of which were considered high quality.

### 2.6 Statistical analysis

Stata software (version 15.1) was used for all statistical analyses. Q and I^2^ statistics (36, 37) were used to evaluate heterogeneity. Significant heterogeneity was indicated by *P* ≤0.05 or I² ≥50%. Subgroup and meta-regression analyses were performed to investigate the potential causes of significant heterogeneity.

Currently, almost all commonly used meta-analysis software allows meta-analysis under two models: the fixed-effects model and the random-effects model. The commonly used selection principle is to select a model according to the level of heterogeneity. However, a few researchers believe this method does not conform to the inherent statistical hypotheses of the model (38). The fixed effects model assumes that all studies in the meta-analysis had the same effect size, whereas the random effects model assumes that different studies had different effect sizes. Although the two models are weighted by the inverse variance method, the fixed effects model only considers the sampling error; therefore, the weight of the large sample study was larger and that of the small sample study was smaller. In the random-effects model, the decisiveness of sample size for weight was relatively weak. The differences in characteristics and sample sizes among the included studies are evident in the data discussed in **Table 1**, indicating the difficulty in maintaining a high degree of consistency. Accordingly, we chose a random-effects model. All included studies were cohort studies, and the data were two-category outcomes. Therefore, we extracted the total number of studies and the number of occurrences in the studies and calculated the ratio of the incidence of outcomes between the intervention and control groups to obtain the RR (a more direct measure than OR). A forest plot was used to represent the meta-analysis results using Stata software.

**Table 1:** Characteristics of the studies that were included.

Study	Country	Study design	Sample size^a^	Male	Median age (IQR)(years)	Tumor type	Diagnostic method of COVID-19	Time^b^	Outcomes	NOS^g^
Hatic H, 2022 (39)	USA	Retrospective	121	70 (57.9%)	63.7^#^	No special	1,2^c^	Within 365 days	Mortality and ICU	9
Isgrò M A,2021 (40)	Italy	Retrospective	885	568 (64.2%)	NA^d^	No special	2	Within 45 days	Infection	7
Luo J,2020 (41)	USA	Retrospective	69	33 (47.8%)	69.0 (31.0–91.0)	Lung cancer	1	Within a median of 45 days	Mortality and ICU	7
Jee J,2021 (42)	USA	Retrospective	608	NA	NA	No special	1	Within 90 days	Respiratory failure or mortality^e^	8
Lièvre A,2020 (43)	France	Retrospective	1289	795 (61.7%)	67.0 (19.0–100.0)	No special	1,2	within 90 days	Mortality	8
Mandala M,2021 (44)	Italy	Prospective	293	182 (62.1%)	NA	No special	1,2	within 90 days	Infection and mortality	7
Gonzalez-Cao M,2022 (45)	Spain	Prospective	150	90 (60.0%)	68.0 (6.0–95.0)	Melanoma	NA	NA	mortality	7
Lara O D,2022 (46)	USA	Retrospective	193	0 (0.0%)	65.0 (54.0–73.0)	Gynecologic cancer	1,2,4	within 90 days	Mortality and ICU	9
Bersanelli M,2020 (47)	Italy	Prospective	955	648 (67.9%)	69.5^#^	No special	1,4	within 90 days	Infection and mortality	7
Calles A,2020 (48)	Spain	Retrospective	242	137 (56.7%)	NA	Lung cancer	1	within 90 days	Infection and mortality	7
Fuentes Antrás J,2020 (49)	Spain	Prospective	73	41 (56.1%)	72.0 (59.0–82.0)	No special	1	NA	Mortality	7
Garassino M C,2020 (50)	Italy	Retrospective	200	141 (70.5%)	68.0 (61.8–75.0)	No special	1,3,4	Within a median of 7 days	Mortality	8
Nie L,2021 (51)	China	Retrospective	45	31 (68.9%)	66.0 (58.0–74.0)	Lung cancer	1	within 28 days	Mortality	7
Nichetti F, 2020 (52)	Italy	Retrospective	1081	491 (45.4%)	63.0 (19.0–91.0)	No special	1	within 54 days	Infection, mortality and ICU	7
Yarza R,2020 (53)	Spain	Prospective	63	34 (54.0%)	NA	No special	1,2,4	within 28 days	ARDS^f^	8

a Sample size means all patients in a study.

b Time is the time of antitumor treatment before SARS-CoV-2 diagnosis.

c 1. RT-PCR; 2. Positive serology test, 3. suspected SARS-CoV-2 infection with symptoms; and 4. radiologic imaging.

d NA means data not available.

e This outcome did not have separate data on mortality; therefore, it was divided into severity.

f ARDS means acute respiratory distress syndrome.

g NOS means The Newcastle-Ottawa Scale.

^#^ Mean age.

Funnel plots were used to calculate publishing bias. Additionally, tests for Begg’s rank correlation and Egger’s linear regression were performed to demonstrate publication bias, with *P <*0.05 indicating significant publication bias. Additionally, sensitivity analysis was performed by eliminating one study at a time to determine whether the outcomes were affected by that particular study.

## 3 Results

### 3.1 Results of the electronic literature search

**Figure 1:**
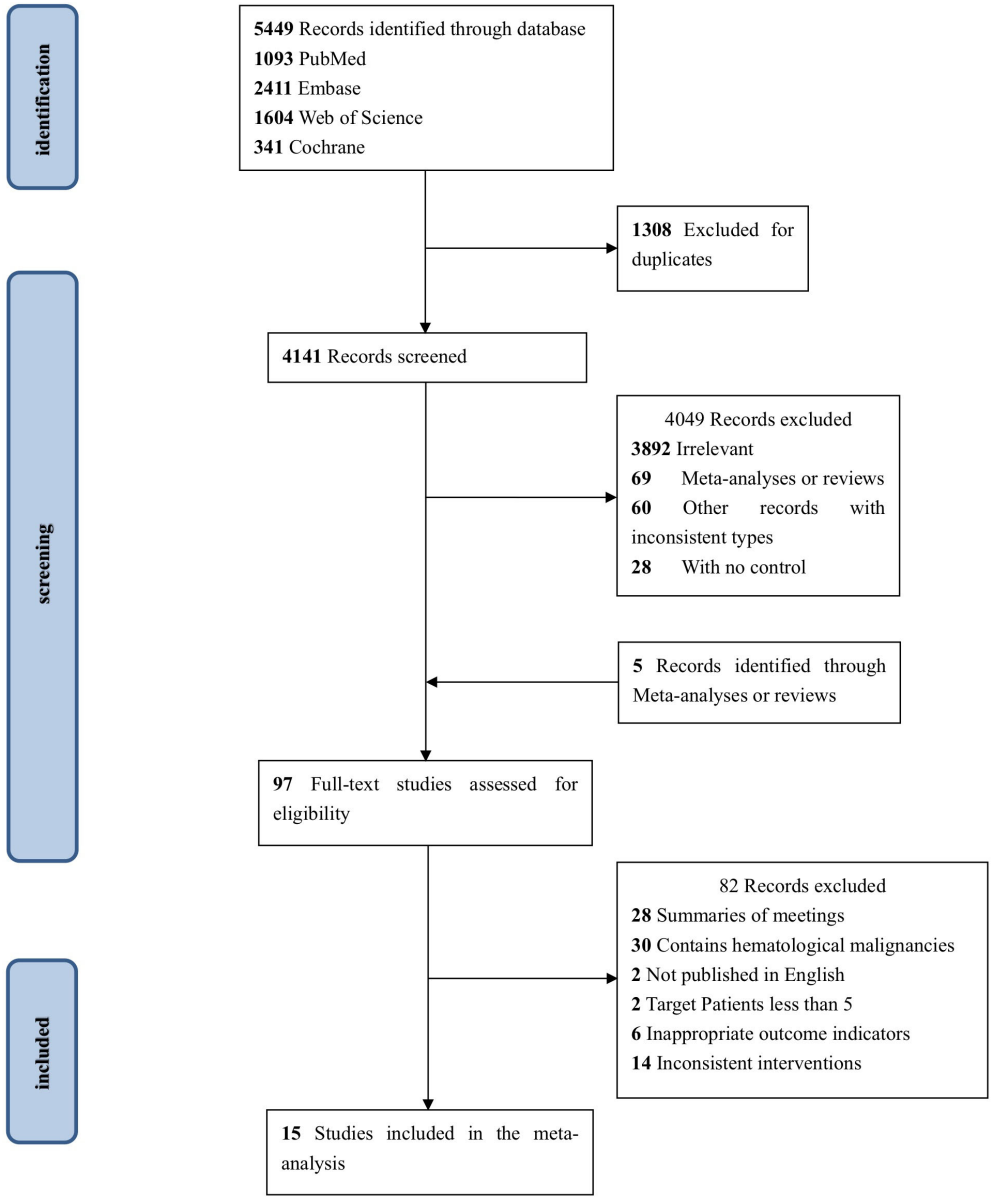
PRISMA flow diagram of study inclusion.

After 5,449 publications were retrieved from the electronic databases, 97 studies were selected for full-text screening based on the eligibility requirements for titles and abstracts. Inconsistency in cancer type was the most common cause of exclusion in the full-text screening. In the end, 15 studies (39–53) were selected, having met all of the inclusion criteria. In **Figure 1** we show the PRISMA diagram used to select the included research.

### 3.2 Study characteristics

The basic characteristics of the included studies are listed in **Table 1**. In total, 6,267 subjects who satisfied the inclusion criteria were included in the analysis. Retrospective cohort studies comprised the majority of the included studies, with five (citations 44, 45, 47, 49, 53) being prospectively designed. Among the 15 studies included, four (39, 41, 42, 46) were performed in the United States, five (40, 44, 47, 50, 52) in Italy, four (45, 48, 49, 53) in Spain, one (51) in China, and one (43) in France. All patients in the 15 studies had solid tumors: three of the studies (41, 48, 51) involved patients with lung cancer, one study (46) included patients with gynecological tumors, and another study (45) included patients with melanoma; the remaining 10 studies were not specific as to cancer type.

In terms of the main outcomes, five studies (40, 44, 47, 48, 52) included the SARS-CoV-2 infection rate, and twelve (39, 41, 43–52) included the mortality rate. As a secondary result, the severity of SARS-CoV-2 was recorded in six of the studies (39, 41, 42, 46, 52, 53).

### 3.3 Impact of past ICIs exposure on SARS-CoV-2 infection in patients with solid cancer

**Figure 2:**
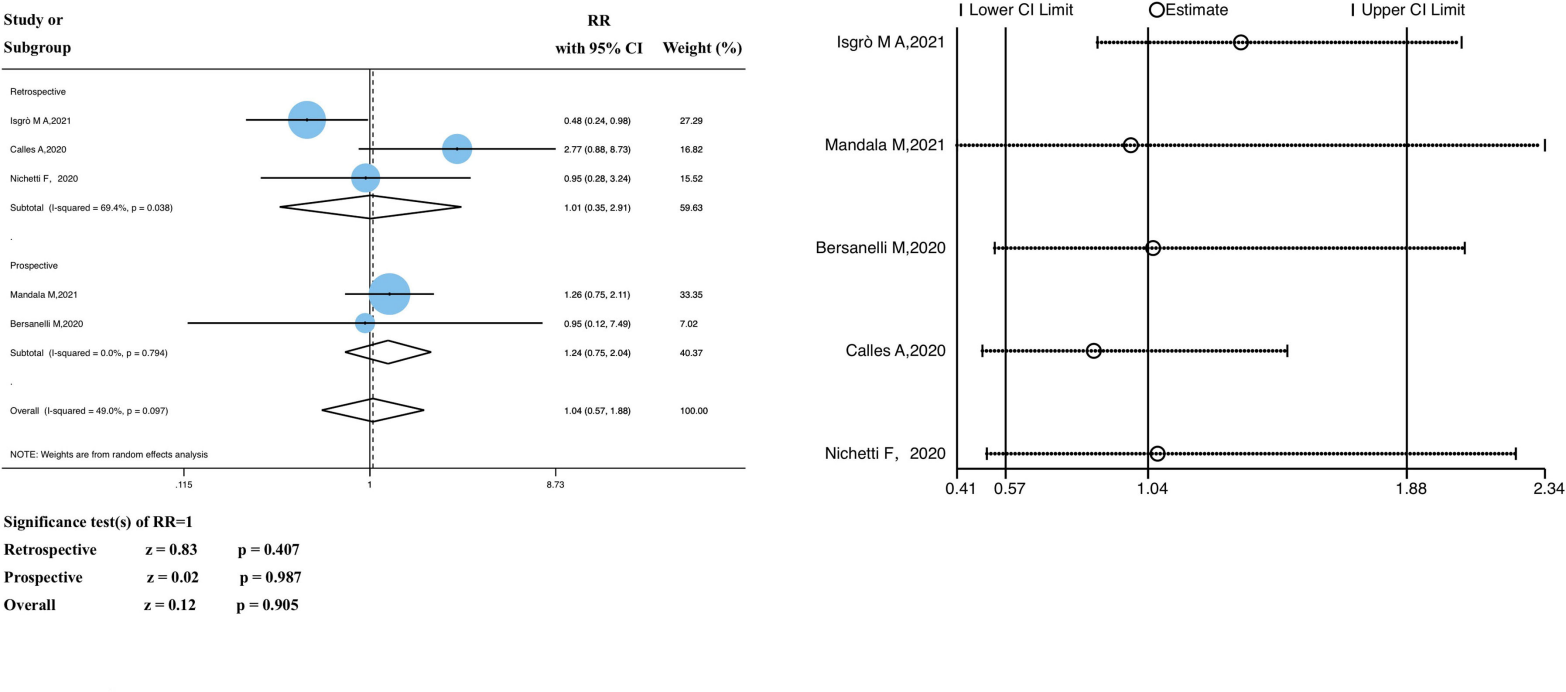
Forest plot of the analysis for the relationship between ICIs and SARS-CoV-2 infection rate, and sensitivity analysis. CI, confidence interval; RR, relative risk.

Five studies analyzed the relationship between ICI treatment and the SARS-CoV-2 infection rate in patients with solid tumors; the pooled data showed no statistically significant differences (RR 1.04, 95% CI 0.57–1.88, z = 0.12; *P* = 0.905) (**Figure 2**).

We divided the study participants into two subgroups (retrospective and prospective) based on the study type. The RR of the retrospective subgroup was 1.01 (95% CI 0.36–2.91, z = 0.83, *P* = 0.407), compared with that of the prospective subgroup, 1.24 (95% CI 0.75–2.04, z = 0.02, *P* = 0.987). In the heterogeneity analysis, there was significant heterogeneity in the retrospective study subgroup (*P* = 0.038, I^2 = ^69.4%); however, the grouping factors were not the source of heterogeneity (*P* = 0.097, I^2 = ^49.0%). No significant heterogeneity was observed. As shown in **Supplementary Material 3**, meta-regression analyses were performed to identify potential sources of significant heterogeneity; there were no significant differences (*P >*0.05).

The funnel plot of the SARS-CoV-2 infection rate showed publication bias (**Supplementary Material 4**); however, further investigation revealed that there was insufficient evidence of significant publication bias (Begg’s test, *P* = 1.000; Egger’s test, *P* = 0.873) (**Supplementary Material 5**). In addition, sensitivity analyses that excluded each included study at a time revealed that the pooled RR values were not significantly influenced by the exclusion of any single study (**Figure 2**). Therefore, we conclude that the findings of this study are satisfactory.

### 3.4 Impact of past ICIs exposure on SARS-CoV-2 mortality in patients with solid cancer

**Figure 3:**
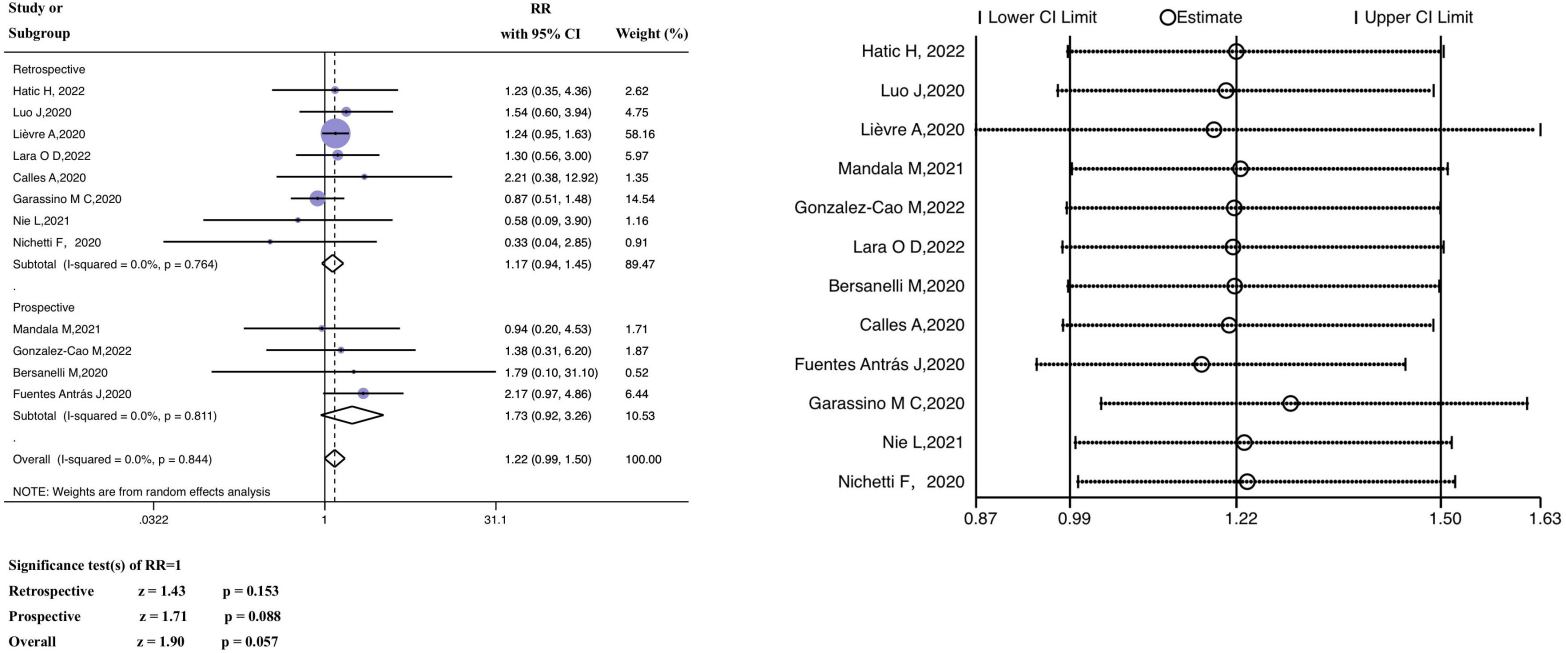
Forest plot of the analysis for the relationship between ICIs and mortality, and sensitivity analysis. CI, confidence interval; RR, relative risk.

Based on the meta-analysis, the pooled estimate RR of patients with past ICI exposure was 1.22 (95% CI 0.99–1.50, z = 1.90, *P* = 0.057) (**Figure 3**), however the RR in the ICI treatment group was not significantly different from the RR in the group that had not received ICI treatment.

A subgroup analysis (retrospective versus prospective) was performed. The average (pooled) RR of the retrospective subgroup was 1.17 (95% CI 0.94–1.45, z =1.43, *P* = 0.153), and the corresponding RR of the prospective subgroup was 1.73 (95% CI 0.92–3.26, z = 1.71, *P* = 0.088). In the heterogeneity analysis, there was no significant heterogeneity in either the retrospective (*P* = 0.764, I^2 = ^0.0%) or prospective study subgroups (*P* = 0.811, I^2 = ^0.0%), and the grouping factors were not the source of heterogeneity (*P* = 0.844, I^2 = ^0.0%). The findings of the meta-regression are discussed in **Supplementary Material 3**. The investigated factors did not show heterogeneity (*P >*0.05).

In examining the data from the Hatic H trial (2022) (39), we discovered that the anti-cancer treatment period before the diagnosis of SARS-CoV-2 was within 365 days; thus, the baseline was uneven compared to other studies. In constructing funnel plots of the included studies, we observed no publication bias, as shown in **Supplementary Material 4**. The results of Egger’s linear regression and Begg’s rank correlation tests are included in **Supplementary Material 5** (Begg’s test, *P* = 0.537; Egger’s test, *P* = 0.881). Furthermore, the average effect size was unaffected by sensitivity analyses that excluded one included study at a time (**Figure 3**). Therefore, the article in question was not deleted from our analysis.

### 3.5 Impact of past ICI exposure on SARS-CoV-2 severity in patients with solid cancers

**Figure 4:**
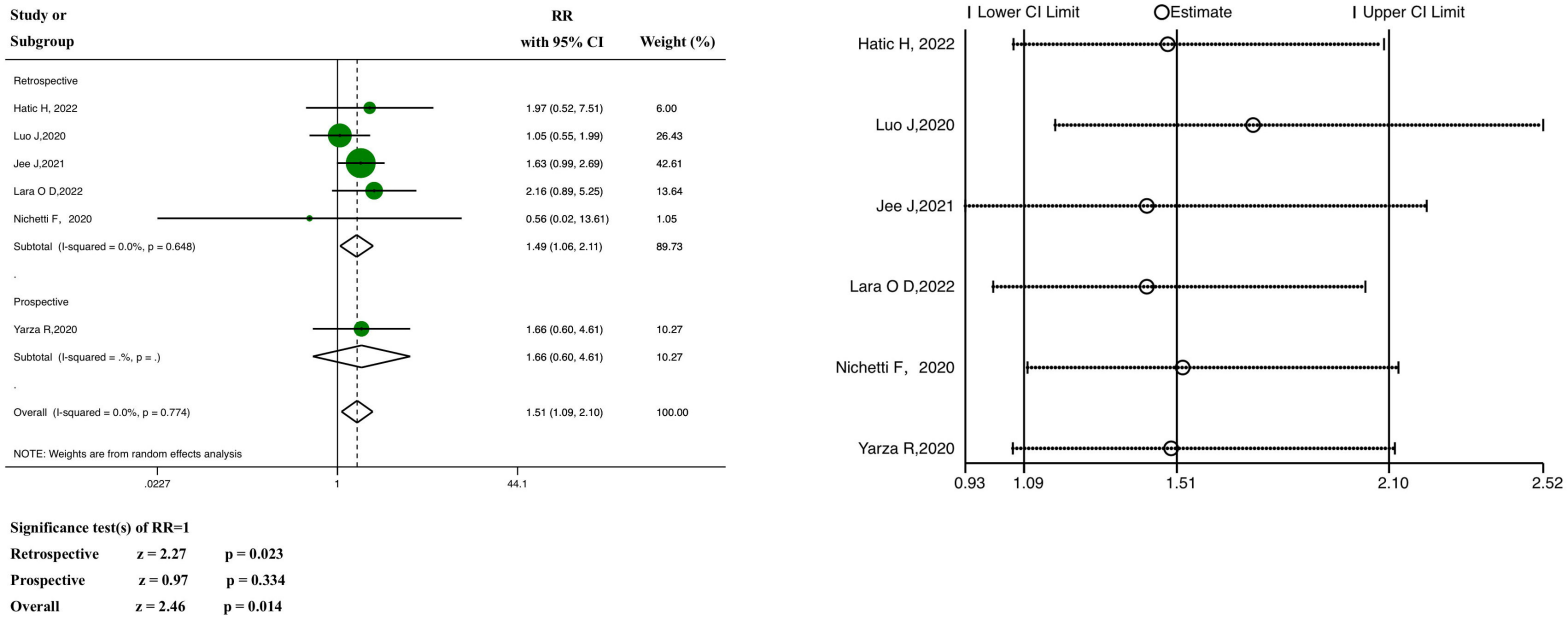
Forest plot of the analysis for the relationship between ICIs and severity, and sensitivity analysis. CI, confidence interval; RR, relative risk.

Information on severe or critical diseases was reported in six of the studies, in which ICI was significantly linked with severe COVID-19 (pooled RR, 1.51; 95% CI, 1.09–2.10, z = 2.46, *P* = 0.014; **Figure 4**).

The results of the subgroup analysis showed that the pooled RR of the retrospective subgroup was 1.49 (95% CI: 1.06–2.11, z =2.27, *P* =0.023), compared with 1.66 in the case of the prospective subgroup (95% CI: 0.60–4.61, z = 0.97, *P* = 0.334; this subgroup included only one study). In the heterogeneity analysis, there was no significant heterogeneity in either the retrospective study subgroup (*P* = 0.648, I^2 = ^0.0%) or the prospective study subgroup (only one study), and the grouping factors were not the source of heterogeneity (*P* = 0.774, I^2 = ^0.0%). Meta-regression analysis (**Supplementary Material 3**) showed no heterogeneity (*P >*0.05).

Neither the funnel plot (**Supplementary Material 4**) nor the tests for significant publication bias (Begg’s test, *P* = 1.000; Egger’s test, *P* = 0.930) (**Supplementary Material 5**) revealed any evidence of potential publication bias. In addition, regardless of excluding any particular study one at a time, the pooled RRs were not significantly influenced by the sensitivity analysis (**Figure 4**).

### 3.6 Cancer type

In terms of tumor types, we included cohort studies on solid tumors, covering lung cancer, gynecological tumors, melanoma, and other types of cancer; however, due to the lack of data in the included studies, we analyzed only the data of patients with lung cancer and observed that there was no significant correlation between the treatment of ICIs and the infection rate (pooled RR, 1.55; 95% CI, 0.48–5.01, z = 0.73, *P* = 0.468; heterogeneity test, *P* = 0.039, I^2 = ^69.1%) and the mortality rate (pooled RR, 1.56; 95% CI, 0.63–3.84, z = 0.96, *P* = 0.338; heterogeneity test, *P* = 0.314, I^2 = ^13.7%). The specific data are provided in **Supplementary Material 6**.

In addition, owing to the inclusion of studies from the COVID pandemic period (from 2020 to 2022), we conducted a subgroup analysis of the years. The results showed that, in terms of infection, the pooled estimate RR was 1.04 (95% CI 0.57–1.88, z = 0.12, *P* = 0.905) (**Supplementary Material 7**), which was not statistically significant. The pooled RR of the 2021 subgroup was 0.80 (95% CI 0.31–2.06, z = 0.46, *P* = 0.648), compared with the corresponding value, 1.55, from the 2020 subgroup (95% CI 0.71–3.37, z = 1.11, *P* = 0.269). At the same time, in terms of mortality, the pooled estimate had no statistically significant differences (RR 1.22, 95% CI 0.99–1.50, z = 1.90, *P* = 0.057). The pooled RR of the 2022 subgroup was 1.29 (95% CI 0.69–2.44, z = 0.80, *P* = 0.424); that of the 2021 subgroup was 0.78 (95% CI 0.23–2.60, z = 0.41, *P* = 0.682); and 1.23 in the 2020 subgroup (95% CI 0.99–1.53, z = 1.84, *P* = 0.065).

## 4 Discussion

The outbreak of SARS-CoV-2 has disrupted many aspects of human life, especially health care. A meta-analysis by Muka et al. (54) confirmed that the pandemic had a significant impact on cancer care, including delays in screening, diagnosis, and treatment. In the short term, these results suggest that interventions are needed to mitigate the negative impacts of infectious disease epidemics on cancer care. In the long run, they demonstrated the importance of rigorous systematic reviews in guiding decision-making. To the best of our knowledge, this systematic review and meta-analysis is the first to assess the association between ICIs and SARS-CoV-2 infection and prognosis in patients with solid cancers. The analysis was performed on pooled data from 15 cohort studies. We used a random-effects model to fit the actual sampling distribution as much as possible and extended the conclusion to a wide range of scenarios.

In addition to cancer immunosurveillance, the immune system plays a crucial role in protecting against pathogens, bacteria, viruses, and fungi. T cells are continually subjected to antigens, including recurrent viral infections, which contribute to T cell exhaustion. This implies that ICIs may increase CD8+ T cell proliferative capabilities by obstructing CTLA4 and PD-1/PD-L1-mediated signaling pathways, theoretically lowering the viral load (55). PD-1-targeted treatment inhibits tumor growth and lowers viral load in various cancer and persistent infection mouse models (56). The same observations have been made in the case of non-human primates, Yatim et al. (57) provided evidence that ICI during COVID-19 enhanced T-cell immunity without exacerbating inflammation. Additionally, patients with cancer receiving ICIs can recover their immunocompetence after contracting human immunodeficiency virus (HIV), hepatitis B, or hepatitis C viruses, indicating that they may be significantly more immunocompetent than other patients with cancer receiving chemotherapy or radiation therapy (58). Consequently, several therapeutic trials, including the use of immune checkpoint blockade in treating recurrent viral infections, have been designed and administered to patients with cancer (59).

The main outcomes of our study support the judicious use of ICIs in treating patients with cancer who have been previously exposed to SARS-CoV-2. Our main outcomes showed that prior exposure of patients with solid cancers to ICIs was not linked to a higher risk of SARS-CoV-2 infection or mortality (*P >*0.05). However, the analysis showed that (based on six of the selected studies), the use of ICIs may aggravate the condition of SARS-CoV-2 in patients with solid cancer and increase the risk of adverse clinical outcomes (*P <*0.05). Considering these results together, the conclusion is that practitioners need to carefully evaluate the benefits and drawbacks of the use of ICIs in patients with solid tumors, considering their history of SARS-CoV-2 infection, and proceed with caution.

To address potential heterogeneity in the selected study population, we conducted a subgroup analysis of the study types and observed that the differences between prospective and retrospective studies were not a source of heterogeneity. In addition, considering the possible impact of the different prevalence rates of SARS-CoV-2 from 2020 to 2022 on the results, we conducted an analysis of subgroups by year and observed no significant correlation.

Among all solid tumors, lung cancer is the most dangerous to patients who had been infected with SARS-CoV-2 prior to their cancer treatment (60). Patients with lung tumors tend to have relatively poor physical status. Smoking habits, and multiple comorbid diseases often present with comorbid lung disease and poor lung function (61) and are predisposed to respiratory infections. Therefore, we constructed a forest plot for a small portion of the study cohort, those patients with lung cancer. Our results showed no significant correlation between ICIs treatment and the SARS-CoV-2 infection and mortality rates of patients with lung cancer. SARS-CoV-2 does not tend to be more severe in lung cancer cases. In future studies, a greater range of literature with adequate and transparent reporting of tumor types should be included to confirm the applicability of our findings.

Our study observed that ICIs were not associated with a higher SARS-CoV-2 infection rate or mortality in patients with solid tumors, which is inconsistent with previous research results (62). We note that previous studies have combined hematological malignancies as one of the possible reasons for the difference in results. In contrast to most solid tumors, hematological malignancies are highly susceptible to interactions with immune cells and are responsive to ongoing systemic inflammatory reactions (63). Moreover, whereas patients with solid tumors appear to no longer be at risk of SARS-CoV-2-associated immune dysregulation compared to the general population, patients with hematological cancer display complicated immunological consequences of SARS-CoV-2 exposure (64) and tend to have poor prognosis (65, 66). For this reason, we analyzed data from solid tumor cases, rather than other forms of cancer.

Our findings are supported by those of a few previous studies. In a systematic analysis, Lazarus et al. (29) included 11 trials with a total of 2,826 patients with cancer infected with COVID-19. The authors observed that moderate-to-high-quality evidence for ICIs was not associated with an increased mortality risk. According to Liu et al. (30), who analyzed data from 5,121 patients with cancer and COVID-19, there was no discernible difference in mortality between groups of patients undergoing anti-tumor therapy (including immunotherapy) and those who were not.

This systematic review has several strengths. To the best of our knowledge, this is the first comprehensive analysis of SARS-CoV-2 infection and prognosis in patients with solid tumors receiving ICIs. Second, it incorporates a sizable number of studies not covered in earlier evaluations, making it the most thorough and reliable body of information available to date. Third, minimal or non-existent publication bias and inter-study increases our confidence in the findings.

Our analysis has several limitations. First, although our dataset included different types of cancer, we chose to separate the data associated with cases of lung cancer (and produced a forest plot from this data). The results suggest that there is no significant correlation between ICI treatment and the SARS-CoV-2 infection and mortality rates in patients with lung cancer. However, because of the small sample size and relative paucity of studies, we cannot guarantee the accuracy of the results. Subgroup analysis based on cancer type was not performed because other tumor types could not be separated from the valid data. In terms of differences in cancer staging, we did not find the corresponding outcome indicator data in the literature. Moreover, there will be more research on a certain type or stage of cancer in this field in the future. Second, we set the control group as patients receiving other antitumor treatments (other than ICI). Differences exist in the modes of action between the antitumor treatments, which may have adversely affected the results. Currently, the literature is not sufficient to allow a robust comparison of a particular anti-tumor treatment with ICIs. In the future, we will actively conduct more narrowly defined grouping studies while expanding the dataset to include a greater number of cases that meet the inclusion criteria.

## 5 Conclusion

This is the first meta-analysis in which the possible association between ICI treatment and SARS-CoV-2 infection and prognosis was evaluated using retrospective data from patients with solid cancers. Our main findings are that the prior exposure of patients with solid cancer to ICIs was not linked to a higher risk of SARS-CoV-2 infection or mortality. However, in the secondary outcomes, ICI use was significantly associated with a greater probability of severe/critical disease in individuals with solid tumors before SARS-CoV-2 diagnosis. Therefore, we believe that in the current environment, the use of ICIs is not categorically disadvantageous to patients with solid tumors, but caution must be exercised.

## Data availability statement

The original contributions presented in the study are included in the article/**Supplementary material**. Further inquiries can be directed to the corresponding author.

## Author contributions

LS: Conceptualization, Formal analysis, Investigation, Software, Validation, Writing – original draft. FZ: Conceptualization, Formal analysis, Investigation, Software, Validation, Writing – review & editing. YX: Conceptualization, Validation, Writing – review & editing. SC: Project administration, Validation, Writing – review & editing. QS: Conceptualization, Supervision, Writing – review & editing.
