# Supplementary material for: Association of immune checkpoint inhibitors with SARS-CoV-2 infection rate and prognosis in patients with solid tumors: a systematic review and meta-analysis
Source: Front Immunol. 2024 Jun 3;15:1259112. doi: 10.3389/fimmu.2024.1259112 (PMC11180804; doi:10.3389/fimmu.2024.1259112)
Supplement: Supplementary file 1 [file DataSheet_1.docx]

Supplementary Material

# Supplementary Data

**Supplementary Material 1** Search strategies for PubMed, Embase, Web of Science and Cochrane database

**PubMed: 1,093 results**

(((("COVID-19"[Mesh]) OR (COVID 19[Title/Abstract]) OR (2019-nCoV Infection[Title/Abstract]) OR (2019 nCoV Infection[Title/Abstract]) OR (2019-nCoV Infections[Title/Abstract]) OR (Infection, 2019-nCoV[Title/Abstract]) OR (SARS-CoV-2 Infection[Title/Abstract]) OR (Infection, SARS-CoV-2[Title/Abstract]) OR (SARS CoV 2 Infection[Title/Abstract]) OR (SARS-CoV-2 Infections[Title/Abstract]) OR (2019 Novel Coronavirus Disease[Title/Abstract]) OR (2019 Novel Coronavirus Infection[Title/Abstract]) OR (COVID-19 Virus Infection[Title/Abstract]) OR (COVID 19 Virus Infection[Title/Abstract]) OR (COVID-19 Virus Infections[Title/Abstract]) OR (Infection, COVID-19 Virus[Title/Abstract]) OR (Virus Infection, COVID-19[Title/Abstract]) OR (COVID19[Title/Abstract]) OR (Coronavirus Disease 2019[Title/Abstract]) OR (Disease 2019, Coronavirus[Title/Abstract]) OR (Coronavirus Disease-19[Title/Abstract]) OR (Coronavirus Disease 19[Title/Abstract]) OR (Severe Acute Respiratory Syndrome Coronavirus 2 Infection[Title/Abstract]) OR (COVID-19 Virus Disease[Title/Abstract]) OR (COVID 19 Virus Disease[Title/Abstract]) OR (COVID-19 Virus Diseases[Title/Abstract]) OR (Disease, COVID-19 Virus[Title/Abstract]) OR (Virus Disease, COVID-19[Title/Abstract]) OR (SARS Coronavirus 2 Infection[Title/Abstract]) OR (2019-nCoV Disease[Title/Abstract]) OR (2019 nCoV Disease[Title/Abstract]) OR (2019-nCoV Diseases[Title/Abstract]) OR (Disease, 2019-nCoV[Title/Abstract]) OR (COVID-19 Pandemic[Title/Abstract]) OR (COVID 19 Pandemic[Title/Abstract]) OR (Pandemic, COVID-19[Title/Abstract]) OR (COVID-19 Pandemics[Title/Abstract])) OR (("SARS-CoV-2"[Mesh]) OR (SARS-CoV-2 Virus[Title/Abstract]) OR (SARS CoV 2 Virus[Title/Abstract]) OR (SARS-CoV-2 Viruses[Title/Abstract]) OR (Virus, SARS-CoV-2[Title/Abstract]) OR (2019 Novel Coronavirus[Title/Abstract]) OR (2019 Novel Coronaviruses[Title/Abstract]) OR (Coronavirus, 2019 Novel[Title/Abstract]) OR (Novel Coronavirus, 2019[Title/Abstract]) OR (COVID-19 Virus[Title/Abstract]) OR (COVID 19 Virus[Title/Abstract]) OR (COVID-19 Viruses[Title/Abstract]) OR (Virus, COVID-19[Title/Abstract]) OR (Wuhan Coronavirus[Title/Abstract]) OR (Coronavirus, Wuhan[Title/Abstract]) OR (COVID19 Virus[Title/Abstract]) OR (COVID19 Viruses[Title/Abstract]) OR (Virus, COVID19[Title/Abstract]) OR (Viruses, COVID19[Title/Abstract]) OR (Coronavirus Disease 2019 Virus[Title/Abstract]) OR (Severe Acute Respiratory Syndrome Coronavirus 2[Title/Abstract]) OR (SARS Coronavirus 2[Title/Abstract]) OR (Coronavirus 2, SARS[Title/Abstract]) OR (2019-nCoV[Title/Abstract]) OR (Wuhan Seafood Market Pneumonia Virus[Title/Abstract]))) AND (("Neoplasms"[Mesh]) OR (Tumor[Title/Abstract]) OR (Neoplasm[Title/Abstract]) OR (Tumors[Title/Abstract]) OR (Neoplasia[Title/Abstract]) OR (Neoplasias[Title/Abstract]) OR (Cancer[Title/Abstract]) OR (Cancers[Title/Abstract]) OR (Malignant Neoplasm[Title/Abstract]) OR (Malignancy[Title/Abstract]) OR (Malignancies[Title/Abstract]) OR (Malignant Neoplasms[Title/Abstract]) OR (Neoplasm, Malignant[Title/Abstract]) OR (Neoplasms, Malignant[Title/Abstract]) OR (Benign Neoplasms[Title/Abstract]) OR (Benign Neoplasm[Title/Abstract]) OR (Neoplasms, Benign[Title/Abstract]) OR (Neoplasm, Benign[Title/Abstract]))) AND (((("Immunotherapy"[Mesh]) OR (Immunotherapies[Title/Abstract])) OR (("Immune Checkpoint Inhibitors"[Mesh]) OR (Checkpoint Inhibitors, Immune[Title/Abstract]) OR (Immune Checkpoint Inhibitor[Title/Abstract]) OR (Checkpoint Inhibitor, Immune[Title/Abstract]) OR (Immune Checkpoint Blockers[Title/Abstract]) OR (Checkpoint Blockers, Immune[Title/Abstract]) OR (Immune Checkpoint Blockade[Title/Abstract]) OR (Checkpoint Blockade, Immune[Title/Abstract]) OR (Immune Checkpoint Inhibition[Title/Abstract]) OR (Checkpoint Inhibition, Immune[Title/Abstract]) OR (PD-L1 Inhibitors[Title/Abstract]) OR (PD L1 Inhibitors[Title/Abstract]) OR (PD-L1 Inhibitor[Title/Abstract]) OR (PD L1 Inhibitor[Title/Abstract]) OR (Programmed Death-Ligand 1 Inhibitors[Title/Abstract]) OR (Programmed Death Ligand 1 Inhibitors[Title/Abstract]) OR (PD-1-PD-L1 Blockade[Title/Abstract]) OR (Blockade, PD-1-PD-L1[Title/Abstract]) OR (PD 1 PD L1 Blockade[Title/Abstract]) OR (CTLA-4 Inhibitors[Title/Abstract]) OR (CTLA 4 Inhibitors[Title/Abstract]) OR (CTLA-4 Inhibitor[Title/Abstract]) OR (CTLA 4 Inhibitor[Title/Abstract]) OR (Cytotoxic T-Lymphocyte-Associated Protein 4 Inhibitors[Title/Abstract]) OR (Cytotoxic T Lymphocyte Associated Protein 4 Inhibitors[Title/Abstract]) OR (Cytotoxic T-Lymphocyte-Associated Protein 4 Inhibitor[Title/Abstract]) OR (Cytotoxic T Lymphocyte Associated Protein 4 Inhibitor[Title/Abstract]) OR (PD-1 Inhibitors[Title/Abstract]) OR (PD 1 Inhibitors[Title/Abstract]) OR (PD-1 Inhibitor[Title/Abstract]) OR (Inhibitor, PD-1[Title/Abstract]) OR (PD 1 Inhibitor[Title/Abstract]) OR (Programmed Cell Death Protein 1 Inhibitor[Title/Abstract]) OR (Programmed Cell Death Protein 1 Inhibitors[Title/Abstract]))) OR (("Nivolumab"[Mesh]) OR (Opdivo[Title/Abstract]) OR (ONO-4538[Title/Abstract]) OR (ONO 4538[Title/Abstract]) OR (ONO4538[Title/Abstract]) OR (MDX-1106[Title/Abstract]) OR (MDX 1106[Title/Abstract]) OR (MDX1106[Title/Abstract]) OR (BMS-936558[Title/Abstract]) OR (BMS 936558[Title/Abstract]) OR (BMS936558[Title/Abstract]) OR (Pembrolizumab[Title/Abstract]) OR (SCH-900475[Title/Abstract]) OR (lambrolizumab[Title/Abstract]) OR (MK-3475[Title/Abstract]) OR (Keytruda[Title/Abstract]) OR (Cemiplimab[Title/Abstract]) OR (REGN2810[Title/Abstract]) OR (Camrelizumab[Title/Abstract]) OR (carrelizumab[Title/Abstract]) OR (SHR-1210[Title/Abstract]) OR (SHR 1210[Title/Abstract]) OR (sintilimab[Title/Abstract]) OR (IBI 308[Title/Abstract]) OR (IBI308[Title/Abstract]) OR (IBI-308[Title/Abstract]) OR (toripalimab[Title/Abstract]) OR (JS001[Title/Abstract]) OR (Tislelizumab[Title/Abstract]) OR (BGB-A317[Title/Abstract]) OR (Penpulimab[Title/Abstract]) OR (AK105[Title/Abstract]) OR (zimberelimab[Title/Abstract]) OR (GLS-010[Title/Abstract]) OR (serplulimab[Title/Abstract]) OR (HLX10[Title/Abstract]) OR (Pucotenlimab[Title/Abstract]) OR (HX008[Title/Abstract]) OR (Atezolimumab[Title/Abstract]) OR (anti-PDL1[Title/Abstract]) OR (MPDL3280A[Title/Abstract]) OR (MPDL-3280A[Title/Abstract]) OR (Tecentriq[Title/Abstract]) OR (RG7446[Title/Abstract]) OR (RG-7446[Title/Abstract]) OR (Durvalumab[Title/Abstract]) OR (MEDI4736[Title/Abstract]) OR (MEDI-4736[Title/Abstract]) OR (Imfinzi[Title/Abstract]) OR (Avelumab[Title/Abstract]) OR (MSB-0010682[Title/Abstract]) OR (MSB0010682[Title/Abstract]) OR (bavencio[Title/Abstract]) OR (MSB0010718C[Title/Abstract]) OR (MSB-0010718C[Title/Abstract]) OR (Envafolimab[Title/Abstract]) OR (KN035[Title/Abstract]) OR (Sugemalimab[Title/Abstract]) OR (CS1001[Title/Abstract]) OR (Ipilimumab[Title/Abstract]) OR (Anti-CTLA-4 MAb Ipilimumab[Title/Abstract]) OR (Anti CTLA 4 MAb Ipilimumab[Title/Abstract]) OR (Ipilimumab, Anti-CTLA-4 MAb[Title/Abstract]) OR (Yervoy[Title/Abstract]) OR (MDX 010[Title/Abstract]) OR (MDX010[Title/Abstract]) OR (MDX-010[Title/Abstract]) OR (MDX-CTLA-4[Title/Abstract]) OR (MDX CTLA 4[Title/Abstract]) OR (tremelimumab[Title/Abstract]) OR (ticilimumab[Title/Abstract]) OR (CP 675[Title/Abstract]) OR (CP675 cpd[Title/Abstract]) OR (CP-675[Title/Abstract]) OR (CP-675,206[Title/Abstract]) OR (CP-675206[Title/Abstract]) OR (CP675206[Title/Abstract]) OR (CP 675206[Title/Abstract]) OR (Candonilimab[Title/Abstract]) OR (AK104[Title/Abstract])))

**Embase: 2,411 results**

#1 ('covid 19':ti,ab,kw OR '2019-ncov infection':ti,ab,kw OR '2019 ncov infection':ti,ab,kw OR '2019-ncov infections':ti,ab,kw OR 'infection, 2019-ncov':ti,ab,kw OR 'sars-cov-2 infection':ti,ab,kw OR 'infection, sars-cov-2':ti,ab,kw OR 'sars cov 2 infection':ti,ab,kw OR 'sars-cov-2 infections':ti,ab,kw OR '2019 novel coronavirus disease':ti,ab,kw OR '2019 novel coronavirus infection':ti,ab,kw OR 'covid-19 virus infection':ti,ab,kw OR 'covid 19 virus infection':ti,ab,kw OR 'covid-19 virus infections':ti,ab,kw OR 'infection, covid-19 virus':ti,ab,kw OR 'virus infection, covid-19':ti,ab,kw OR 'covid19':ti,ab,kw OR 'coronavirus disease 2019':ti,ab,kw OR 'disease 2019, coronavirus':ti,ab,kw OR 'coronavirus disease-19':ti,ab,kw OR 'coronavirus disease 19':ti,ab,kw OR 'severe acute respiratory syndrome coronavirus 2 infection':ti,ab,kw OR 'covid-19 virus disease':ti,ab,kw OR 'covid 19 virus disease':ti,ab,kw OR 'covid-19 virus diseases':ti,ab,kw OR 'disease, covid-19 virus':ti,ab,kw OR 'virus disease, covid-19':ti,ab,kw OR 'sars coronavirus 2 infection':ti,ab,kw OR '2019-ncov disease':ti,ab,kw OR '2019 ncov disease':ti,ab,kw OR '2019-ncov diseases':ti,ab,kw OR 'disease, 2019-ncov':ti,ab,kw OR 'covid-19 pandemic':ti,ab,kw OR 'covid 19 pandemic':ti,ab,kw OR 'pandemic, covid-19':ti,ab,kw OR 'covid-19 pandemics':ti,ab,kw OR 'sars cov 2':ab,kw,ti OR 'sars-cov-2 virus':ab,kw,ti OR 'sars cov 2 virus':ab,kw,ti OR 'sars-cov-2 viruses':ab,kw,ti OR 'virus, sars-cov-2':ab,kw,ti OR '2019 novel coronavirus':ab,kw,ti OR '2019 novel coronaviruses':ab,kw,ti OR 'coronavirus, 2019 novel':ab,kw,ti OR 'novel coronavirus, 2019':ab,kw,ti OR 'covid-19 virus':ab,kw,ti OR 'covid 19 virus':ab,kw,ti OR 'covid-19 viruses':ab,kw,ti OR 'virus, covid-19':ab,kw,ti OR 'wuhan coronavirus':ab,kw,ti OR 'coronavirus, wuhan':ab,kw,ti OR 'covid19 virus':ab,kw,ti OR 'covid19 viruses':ab,kw,ti OR 'virus, covid19':ab,kw,ti OR 'viruses, covid19':ab,kw,ti OR 'coronavirus disease 2019 virus':ab,kw,ti OR 'severe acute respiratory syndrome coronavirus 2':ab,kw,ti OR 'sars coronavirus 2':ab,kw,ti OR 'coronavirus 2, sars':ab,kw,ti OR '2019-ncov':ab,kw,ti OR 'wuhan seafood market pneumonia virus':ab,kw,ti OR 'coronavirus disease 2019'/exp OR 'severe acute respiratory syndrome coronavirus 2'/exp)

#2 ('tumor':ab,kw,ti OR 'neoplasm':ab,kw,ti OR 'tumors':ab,kw,ti OR 'neoplasia':ab,kw,ti OR 'neoplasias':ab,kw,ti OR 'cancer':ab,kw,ti OR 'cancers':ab,kw,ti OR 'malignant neoplasm':ab,kw,ti OR 'malignancy':ab,kw,ti OR 'malignancies':ab,kw,ti OR 'malignant neoplasms':ab,kw,ti OR 'neoplasm, malignant':ab,kw,ti OR 'neoplasms, malignant':ab,kw,ti OR 'benign neoplasms':ab,kw,ti OR 'benign neoplasm':ab,kw,ti OR 'neoplasms, benign':ab,kw,ti OR 'neoplasm, benign':ab,kw,ti OR 'neoplasm'/exp OR 'carcinoma'/exp OR 'malignant neoplasm'/exp)

#3 ('immunotherapy'/exp OR 'immunotherapies':ab,kw,ti OR 'immune checkpoint inhibitor'/exp OR 'checkpoint inhibitors, immune':ab,kw,ti OR 'immune checkpoint inhibitor':ab,kw,ti OR 'checkpoint inhibitor, immune':ab,kw,ti OR 'immune checkpoint blockers':ab,kw,ti OR 'checkpoint blockers, immune':ab,kw,ti OR 'immune checkpoint blockade':ab,kw,ti OR 'checkpoint blockade, immune':ab,kw,ti OR 'immune checkpoint inhibition':ab,kw,ti OR 'checkpoint inhibition, immune':ab,kw,ti OR 'pd-l1 inhibitors':ab,kw,ti OR 'pd l1 inhibitors':ab,kw,ti OR 'pd-l1 inhibitor':ab,kw,ti OR 'pd l1 inhibitor':ab,kw,ti OR 'programmed death-ligand 1 inhibitors':ab,kw,ti OR 'programmed death ligand 1 inhibitors':ab,kw,ti OR 'pd-1-pd-l1 blockade':ab,kw,ti OR 'blockade, pd-1-pd-l1':ab,kw,ti OR 'pd 1 pd l1 blockade':ab,kw,ti OR 'ctla-4 inhibitors':ab,kw,ti OR 'ctla 4 inhibitors':ab,kw,ti OR 'ctla-4 inhibitor':ab,kw,ti OR 'ctla 4 inhibitor':ab,kw,ti OR 'cytotoxic t-lymphocyte-associated protein 4 inhibitors':ab,kw,ti OR 'cytotoxic t lymphocyte associated protein 4 inhibitors':ab,kw,ti OR 'cytotoxic t-lymphocyte-associated protein 4 inhibitor':ab,kw,ti OR 'cytotoxic t lymphocyte associated protein 4 inhibitor':ab,kw,ti OR 'pd-1 inhibitors':ab,kw,ti OR 'pd 1 inhibitors':ab,kw,ti OR 'pd-1 inhibitor':ab,kw,ti OR 'inhibitor, pd-1':ab,kw,ti OR 'pd 1 inhibitor':ab,kw,ti OR 'programmed cell death protein 1 inhibitor':ab,kw,ti OR 'programmed cell death protein 1 inhibitors':ab,kw,ti OR 'cemiplimab'/exp OR 'ticilimumab'/exp OR 'nivolumab':ab,kw,ti OR 'opdivo':ab,kw,ti OR 'ono-4538':ab,kw,ti OR 'ono 4538':ab,kw,ti OR 'ono4538':ab,kw,ti OR 'mdx-1106':ab,kw,ti OR 'mdx 1106':ab,kw,ti OR 'mdx1106':ab,kw,ti OR 'bms-936558':ab,kw,ti OR 'bms 936558':ab,kw,ti OR 'bms936558':ab,kw,ti OR 'pembrolizumab':ab,kw,ti OR 'sch-900475':ab,kw,ti OR 'lambrolizumab':ab,kw,ti OR 'mk-3475':ab,kw,ti OR 'keytruda':ab,kw,ti OR 'cemiplimab':ab,kw,ti OR 'regn2810':ab,kw,ti OR 'camrelizumab':ab,kw,ti OR 'carrelizumab':ab,kw,ti OR 'shr-1210':ab,kw,ti OR 'shr 1210':ab,kw,ti OR 'sintilimab':ab,kw,ti OR 'ibi 308':ab,kw,ti OR 'ibi308':ab,kw,ti OR 'ibi-308':ab,kw,ti OR 'toripalimab':ab,kw,ti OR 'js001':ab,kw,ti OR 'tislelizumab':ab,kw,ti OR 'bgb-a317':ab,kw,ti OR 'penpulimab':ab,kw,ti OR 'ak105':ab,kw,ti OR 'zimberelimab':ab,kw,ti OR 'gls-010':ab,kw,ti OR 'serplulimab':ab,kw,ti OR 'hlx10':ab,kw,ti OR 'pucotenlimab':ab,kw,ti OR 'hx008':ab,kw,ti OR 'atezolimumab':ab,kw,ti OR 'anti-pdl1':ab,kw,ti OR 'mpdl3280a':ab,kw,ti OR 'mpdl-3280a':ab,kw,ti OR 'tecentriq':ab,kw,ti OR 'rg7446':ab,kw,ti OR 'rg-7446':ab,kw,ti OR 'durvalumab':ab,kw,ti OR 'medi4736':ab,kw,ti OR 'medi-4736':ab,kw,ti OR 'imfinzi':ab,kw,ti OR 'avelumab':ab,kw,ti OR 'msb-0010682':ab,kw,ti OR 'msb0010682':ab,kw,ti OR 'bavencio':ab,kw,ti OR 'msb0010718c':ab,kw,ti OR 'msb-0010718c':ab,kw,ti OR 'envafolimab':ab,kw,ti OR 'kn035':ab,kw,ti OR 'sugemalimab':ab,kw,ti OR 'cs1001':ab,kw,ti OR 'ipilimumab':ab,kw,ti OR 'anti-ctla-4 mab ipilimumab':ab,kw,ti OR 'anti ctla 4 mab ipilimumab':ab,kw,ti OR 'ipilimumab, anti-ctla-4 mab':ab,kw,ti OR 'yervoy':ab,kw,ti OR 'mdx 010':ab,kw,ti OR 'mdx010':ab,kw,ti OR 'mdx-010':ab,kw,ti OR 'mdx-ctla-4':ab,kw,ti OR 'mdx ctla 4':ab,kw,ti OR 'tremelimumab':ab,kw,ti OR 'ticilimumab':ab,kw,ti OR 'cp 675':ab,kw,ti OR 'cp675 cpd':ab,kw,ti OR 'cp-675':ab,kw,ti OR 'cp-675,206':ab,kw,ti OR 'cp-675206':ab,kw,ti OR 'cp675206':ab,kw,ti OR 'cp 675206':ab,kw,ti OR 'candonilimab':ab,kw,ti OR 'ak104':ab,kw,ti)

#4 #1 AND #2 AND #3

**Web of Science: 1,604 results**

((TS=(("COVID-19") OR (COVID 19) OR (2019-nCoV Infection) OR (2019 nCoV Infection) OR (2019-nCoV Infections) OR (Infection, 2019-nCoV) OR (SARS-CoV-2 Infection) OR (Infection, SARS-CoV-2) OR (SARS CoV 2 Infection) OR (SARS-CoV-2 Infections) OR (2019 Novel Coronavirus Disease) OR (2019 Novel Coronavirus Infection) OR (COVID-19 Virus Infection) OR (COVID 19 Virus Infection) OR (COVID-19 Virus Infections) OR (Infection, COVID-19 Virus) OR (Virus Infection, COVID-19) OR (COVID19) OR (Coronavirus Disease 2019) OR (Disease 2019, Coronavirus) OR (Coronavirus Disease-19) OR (Coronavirus Disease 19) OR (Severe Acute Respiratory Syndrome Coronavirus 2 Infection) OR (COVID-19 Virus Disease) OR (COVID 19 Virus Disease) OR (COVID-19 Virus Diseases) OR (Disease, COVID-19 Virus) OR (Virus Disease, COVID-19) OR (SARS Coronavirus 2 Infection) OR (2019-nCoV Disease) OR (2019 nCoV Disease) OR (2019-nCoV Diseases) OR (Disease, 2019-nCoV) OR (COVID-19 Pandemic) OR (COVID 19 Pandemic) OR (Pandemic, COVID-19) OR (COVID-19 Pandemics) OR ("SARS-CoV-2") OR (SARS-CoV-2 Virus) OR (SARS CoV 2 Virus) OR (SARS-CoV-2 Viruses) OR (Virus, SARS-CoV-2) OR (2019 Novel Coronavirus) OR (2019 Novel Coronaviruses) OR (Coronavirus, 2019 Novel) OR (Novel Coronavirus, 2019) OR (COVID-19 Virus) OR (COVID 19 Virus) OR (COVID-19 Viruses) OR (Virus, COVID-19) OR (Wuhan Coronavirus) OR (Coronavirus, Wuhan) OR (COVID19 Virus) OR (COVID19 Viruses) OR (Virus, COVID19) OR (Viruses, COVID19) OR (Coronavirus Disease 2019 Virus) OR (Severe Acute Respiratory Syndrome Coronavirus 2) OR (SARS Coronavirus 2) OR (Coronavirus 2, SARS) OR (2019-nCoV) OR (Wuhan Seafood Market Pneumonia Virus) )) AND TS=(("Neoplasms") OR (Tumor) OR (Neoplasm) OR (Tumors) OR (Neoplasia) OR (Neoplasias) OR (Cancer) OR (Cancers) OR (Malignant Neoplasm) OR (Malignancy) OR (Malignancies) OR (Malignant Neoplasms) OR (Neoplasm, Malignant) OR (Neoplasms, Malignant) OR (Benign Neoplasms) OR (Benign Neoplasm) OR (Neoplasms, Benign) OR (Neoplasm, Benign))) AND TS=(("Immunotherapy") OR (Immunotherapies) OR ("Immune Checkpoint Inhibitors") OR (Checkpoint Inhibitors, Immune) OR (Immune Checkpoint Inhibitor) OR (Checkpoint Inhibitor, Immune) OR (Immune Checkpoint Blockers) OR (Checkpoint Blockers, Immune) OR (Immune Checkpoint Blockade) OR (Checkpoint Blockade, Immune) OR (Immune Checkpoint Inhibition) OR (Checkpoint Inhibition, Immune) OR (PD-L1 Inhibitors) OR (PD L1 Inhibitors) OR (PD-L1 Inhibitor) OR (PD L1 Inhibitor) OR (Programmed Death-Ligand 1 Inhibitors) OR (Programmed Death Ligand 1 Inhibitors) OR (PD-1-PD-L1 Blockade) OR (Blockade, PD-1-PD-L1) OR (PD 1 PD L1 Blockade) OR (CTLA-4 Inhibitors) OR (CTLA 4 Inhibitors) OR (CTLA-4 Inhibitor) OR (CTLA 4 Inhibitor) OR (Cytotoxic T-Lymphocyte-Associated Protein 4 Inhibitors) OR (Cytotoxic T Lymphocyte Associated Protein 4 Inhibitors) OR (Cytotoxic T-Lymphocyte-Associated Protein 4 Inhibitor) OR (Cytotoxic T Lymphocyte Associated Protein 4 Inhibitor) OR (PD-1 Inhibitors) OR (PD 1 Inhibitors) OR (PD-1 Inhibitor) OR (Inhibitor, PD-1) OR (PD 1 Inhibitor) OR (Programmed Cell Death Protein 1 Inhibitor) OR (Programmed Cell Death Protein 1 Inhibitors) OR ("Nivolumab") OR (opdiv) OR (ONO-4538) OR (ONO 4538) OR (ono4128) OR (MDX-1106) OR (MDX 1106) OR (mgl1106) OR (BMS-936559) OR (BMS 936559) OR (bms936559) OR (Pembrolizumab) OR (SCH-900475) OR (lambroizumab) OR (MK-3475) OR (Keytruda) OR (Cemiplimab) OR (regn2878) OR (Camrelizumab) OR (camrelizumab) OR (SHR-1210) OR (SHR 1210) OR (sintilimab) OR (IBI 308) OR (ibi305) OR (IBI-308) OR (toripalimab) OR (jb001) OR (Tislelizumab) OR (BGB-A317) OR (Penpulimab) OR (ak135) OR (zimberelimab) OR (GLS-010) OR (serplulimab) OR (hox10) OR (Pucotenlimab) OR (hh008) OR (atezolizumab) OR (anti-PDL1) OR (MPDL3280A) OR (MPDL-3280A) OR (thecentric) OR (rg7440) OR (RG-7446) OR (Durvalumab) OR (MEDI4736) OR (MEDI-4736) OR (immirzi) OR (Avelumab) OR (MSB-0010682) OR (msb001018c) OR (balancio) OR (MSB0010718C) OR (MSB-0010718C) OR (Envafolimab) OR (k3035) OR (Sugemalimab) OR (cs1000) OR (Ipilimumab) OR (Anti-CTLA-4 MAb Ipilimumab) OR (Anti CTLA 4 MAb Ipilimumab) OR (Ipilimumab, Anti-CTLA-4 MAb) OR (yzrxoy) OR (MDX 010) OR (mdx210) OR (MDX-010) OR (MDX-CTLA-4) OR (MDX CTLA 4) OR (tremelimumab) OR (tocilizumab) OR (CP 675) OR (c6675 cpd) OR (CP-675) OR (CP-675,206) OR (CP-695206) OR (cp035206) OR (CP 695206) OR (cadonilimab) OR (ak104))

**Cochrane: 341 results**

#1 MeSH descriptor: [COVID-19] explode all trees

#2 MeSH descriptor: [SARS-CoV-2] explode all trees

#3 MeSH descriptor: [Neoplasms] explode all trees

#4 MeSH descriptor: [Immunotherapy] explode all trees

#5 MeSH descriptor: [Immune Checkpoint Inhibitors] explode all trees

#6 MeSH descriptor: [Nivolumab] explode all trees

#7 COVID-19 Pandemic

#8 COVID 19 Pandemic

#9 Pandemic, COVID-19

#10 COVID-19 Pandemics

#11 2019 nCoV Infection

#12 COVID19

#13 COVID-19 Virus Disease

#14 COVID 19 Virus Infection

#15 COVID-19 Virus Infection

#16 SARS-CoV-2 Infection

#17 SARS CoV 2 Infection

#18 Disease, COVID-19 Virus

#19 SARS Coronavirus 2 Infection

#20 SARS-CoV-2 Infections

#21 Virus Disease, COVID-19

#22 Infection, COVID-19 Virus

#23 COVID-19 Virus Diseases

#24 2019 Novel Coronavirus Disease

#25 COVID 19 Virus Disease

#26 COVID-19 Virus Infections

#27 Infection, SARS-CoV-2

#28 Coronavirus Disease 19

#29 Coronavirus Disease 2019

#30 2019 Novel Coronavirus Infection

#31 Coronavirus Disease-19

#32 Disease 2019, Coronavirus

#33 Severe Acute Respiratory Syndrome Coronavirus 2 Infection

#34 2019 nCoV Disease

#35 Virus, COVID-19

#36 Wuhan Seafood Market Pneumonia Virus

#37 COVID-19 Virus

#38 Viruses, COVID19

#39 Wuhan Coronavirus

#40 Virus, COVID19

#41 Coronavirus, 2019 Novel

#42 SARS Coronavirus 2

#43 SARS-CoV-2 Virus

#44 Novel Coronavirus, 2019

#45 SARS-CoV-2 Viruses

#46 COVID19 Viruses

#47 COVID19 Virus

#48 COVID-19 Viruses

#49 Virus, SARS-CoV-2

#50 2019 Novel Coronaviruses

#51 COVID 19 Virus

#52 2019 Novel Coronavirus

#53 Coronavirus, Wuhan

#54 Coronavirus 2, SARS

#55 Severe Acute Respiratory Syndrome Coronavirus 2

#56 Coronavirus Disease 2019 Virus

#57 #1 OR #2 OR #7 OR #8 OR #9 OR #10 OR #11 OR #12 OR #13 OR #14 OR #15 OR #16 OR #17 OR #18 OR #19 OR #20 OR #21 OR #22 OR #23 OR #24 OR #25 OR #26 OR #27 OR #28 OR #29 OR #30 OR #31 OR #32 OR #33 OR #34 OR #35 OR #36 OR #37 OR #38 OR #39 OR #40 OR #41 OR #42 OR #43 OR #44 OR #45 OR #46 OR #47 OR #48 OR #49 OR #50 OR #51 OR #52 OR #53 OR #54 OR #55 OR #56

#58 Immunotherapies

#59 PD L1 Inhibitors

#60 PD L1 Inhibitor

#61 PD-L1 Inhibitors

#62 Programmed Death-Ligand 1 Inhibitors

#63 PD-L1 Inhibitor

#64 Programmed Death Ligand 1 Inhibitors

#65 PD 1 PD L1 Blockade

#66 Checkpoint Blockers, Immune

#67 Checkpoint Inhibitor, Immune

#68 Checkpoint Inhibitors, Immune

#69 Immune Checkpoint Inhibitor

#70 Immune Checkpoint Blockers

#71 CTLA-4 Inhibitor

#72 Cytotoxic T-Lymphocyte-Associated Protein 4 Inhibitor

#73 Cytotoxic T-Lymphocyte-Associated Protein 4 Inhibitors

#74 CTLA 4 Inhibitor

#75 CTLA-4 Inhibitors

#76 CTLA 4 Inhibitors

#77 Cytotoxic T Lymphocyte Associated Protein 4 Inhibitor

#78 Cytotoxic T Lymphocyte Associated Protein 4 Inhibitors

#79 PD-1 Inhibitors

#80 Inhibitor, PD-1

#81 Programmed Cell Death Protein 1 Inhibitor

#82 PD 1 Inhibitor

#83 Programmed Cell Death Protein 1 Inhibitors

#84 PD 1 Inhibitors

#85 PD-1 Inhibitor

#86 Immune Checkpoint Inhibition

#87 Checkpoint Inhibition, Immune

#88 Immune Checkpoint Blockade

#89 Checkpoint Blockade, Immune

#90 ONO 4538

#91 ONO4538

#92 ONO-4538

#93 BMS 936558

#94 BMS-936558

#95 BMS936558

#96 Opdivo

#97 MDX 1106

#98 MDX-1106

#99 MDX1106

#100 Pembrolizumab

#101 SCH-900475

#102 lambrolizumab

#103 MK-3475

#104 Keytruda

#105 Cemiplimab

#106 REGN2810

#107 Camrelizumab

#108 SHR-1210

#109 SHR 1210

#110 sintilimab

#111 IBI 308

#112 IBI308

#113 IBI-308

#114 toripalimab

#115 JS001

#116 Tislelizumab

#117 BGB-A317

#118 Penpulimab

#119 AK105

#120 zimberelimab

#121 GLS-010

#122 serplulimab

#123 HLX10

#124 Pucotenlimab

#125 HX008

#126 anti-PDL1

#127 MPDL3280A

#128 MPDL-3280A

#129 Tecentriq

#130 RG7446

#131 RG-7446

#132 Durvalumab

#133 MEDI4736

#134 MEDI-4736

#135 Imfinzi

#136 Avelumab

#137 MSB-0010682

#138 MSB0010682

#139 bavencio

#140 MSB0010718C

#141 MSB-0010718C

#142 Envafolimab

#143 KN035

#144 Sugemalimab

#145 CS1001

#146 Ipilimumab

#147 Anti-CTLA-4 MAb Ipilimumab

#148 Anti CTLA 4 MAb Ipilimumab

#149 Ipilimumab, Anti-CTLA-4 MAb

#150 Yervoy

#151 MDX 010

#152 MDX010

#153 MDX-010

#154 MDX-CTLA-4

#155 MDX CTLA 4

#156 tremelimumab

#157 ticilimumab

#158 CP 675

#159 CP675 cpd

#160 CP-675

#161 CP-675,206

#162 CP-675206

#163 CP675206

#164 CP 675206

#165 Candonilimab

#166 AK104

#167 #4 OR #5 OR #6 OR #58 OR #59 OR #60 OR #61 OR #62 OR #63 OR #64 OR #65 OR #66 OR #67 OR #68 OR #69 OR #70 OR #71 OR #72 OR #73 OR #74 OR #75 OR #76 OR #77 OR #78 OR #79 OR #80 OR #81 OR #82 OR #83 OR #84 OR #85 OR #86 OR #87 OR #88 OR #89 OR #90 OR #91 OR #92 OR #93 OR #94 OR #95 OR #96 OR #97 OR #98 OR #99 OR #100 OR #101 OR #102 OR #103 OR #104 OR #105 OR #106 OR #107 OR #108 OR #109 OR #110 OR #111 OR #112 OR #113 OR #114 OR #115 OR #116 OR #117 OR #118 OR #119 OR #120 OR #121 OR #122 OR #123 OR #124 OR #125 OR #126 OR #127 OR #128 OR #129 OR #130 OR #131 OR #132 OR #133 OR #134 OR #135 OR #136 OR #137 OR #138 OR #139 OR #140 OR #141 OR #142 OR #143 OR #144 OR #145 OR #146 OR #147 OR #148 OR #149 OR #150 OR #151 OR #152 OR #153 OR #154 OR #155 OR #156 OR #157 OR #158 OR #159 OR #160 OR #161 OR #162 OR #163 OR #164 OR #165 OR #166

#168 #57 AND #167

# Supplementary Figures and Tables

## Supplementary Tables

**Supplementary Material 2** The Newcastle-Ottawa scale for quality assessment of the studies.

| **Study** | **Selection** | | | | **Comparability** | **Outcome** | | | **Total score** |
| --- | --- | --- | --- | --- | --- | --- | --- | --- | --- |
|  | Representativeness | Selection | Ascertainment | outcome |  | Assessment | Follow-up | Adequacy |  |
| Hatic H,2022 | ★ | ★ | ★ | ★ | ★★ | ★ | ★ | ★ | 9 |
| Isgrò M A,2021 | ★ | ★ | ★ | ★ | ★ | ★ |  | ★ | 7 |
| Luo J,2020 | ★ | ★ | ★ | ★ | ★ | ★ | ★ |  | 7 |
| Jee J,2021 | ★ | ★ | ★ | ★ | ★ | ★ | ★ | ★ | 8 |
| Lièvre A,2020 | ★ | ★ | ★ | ★ | ★★ | ★ | ★ |  | 8 |
| Mandala M,2021 | ★ | ★ | ★ |  | ★★ | ★ | ★ |  | 7 |
| Gonzalez-Cao M,2022 | ★ | ★ | ★ |  | ★ | ★ | ★ | ★ | 7 |
| Lara O D,2022 | ★ | ★ | ★ | ★ | ★★ | ★ | ★ | ★ | 9 |
| Bersanelli M,2020 | ★ | ★ | ★ |  | ★ | ★ | ★ | ★ | 7 |
| Calles A,2020 | ★ | ★ | ★ | ★ | ★ | ★ | ★ |  | 7 |
| Fuentes Antrás J,2020 | ★ | ★ | ★ | ★ |  | ★ | ★ | ★ | 7 |
| Garassino M C,2020 | ★ | ★ | ★ | ★ | ★★ | ★ | ★ |  | 8 |
| Nie L,2021 | ★ | ★ | ★ |  | ★ | ★ | ★ | ★ | 7 |
| Nichetti F，2020 | ★ | ★ |  | ★ | ★ | ★ | ★ | ★ | 7 |
| Yarza R,2020 | ★ | ★ | ★ | ★ | ★ | ★ | ★ | ★ | 8 |

**Supplementary Material 3** Meta-regression analysis showing the heterogeneity for the effect of tumor type (possible confounders) on the association between prior exposure to ICIs and infection/prognosis of SARS-CoV-2 in solid cancer patients. (**a**) infection. (**b**) mortality. (**c**) severity.

**a**

**Meta-analysis regression**  No of studies =  **5**

tau^2 method  **reml**

tau^2 estimate =  **0**

Successive values of tau^2 differ by less than 10^-4: convergence achieved

|  | Coef. | Std. Err. | z | P> \|z\| | [95% Conf. Interval] | |
| --- | --- | --- | --- | --- | --- | --- |
| _cons | **.1637315** | **.5864232** | **0.28** | **0.798** | **-1.702529** | **2.029992** |

**b**

**Meta-analysis regression**  No of studies =  **12**

tau^2 method  **reml**

tau^2 estimate =  **0**

Successive values of tau^2 differ by less than 10^-4: convergence achieved

|  | Coef. | Std. Err. | z | P> \|z\| | [95% Conf. Interval] | |
| --- | --- | --- | --- | --- | --- | --- |
| _cons | **.5498475** | **.3222937** | **1.71** | **0.088** | **-.0818366** | **1.181532** |

**c**

**Meta-analysis regression**  No of studies =  **6**

tau^2 method  **reml**

tau^2 estimate =  **0**

Successive values of tau^2 differ by less than 10^-4: convergence achieved

|  | Coef. | Std. Err. | z | P> \|z\| | [95% Conf. Interval] | |
| --- | --- | --- | --- | --- | --- | --- |
| _cons | **.504556** | **.5223014** | **0.97** | **0.389** | **-.9455851** | **1.954697** |

##
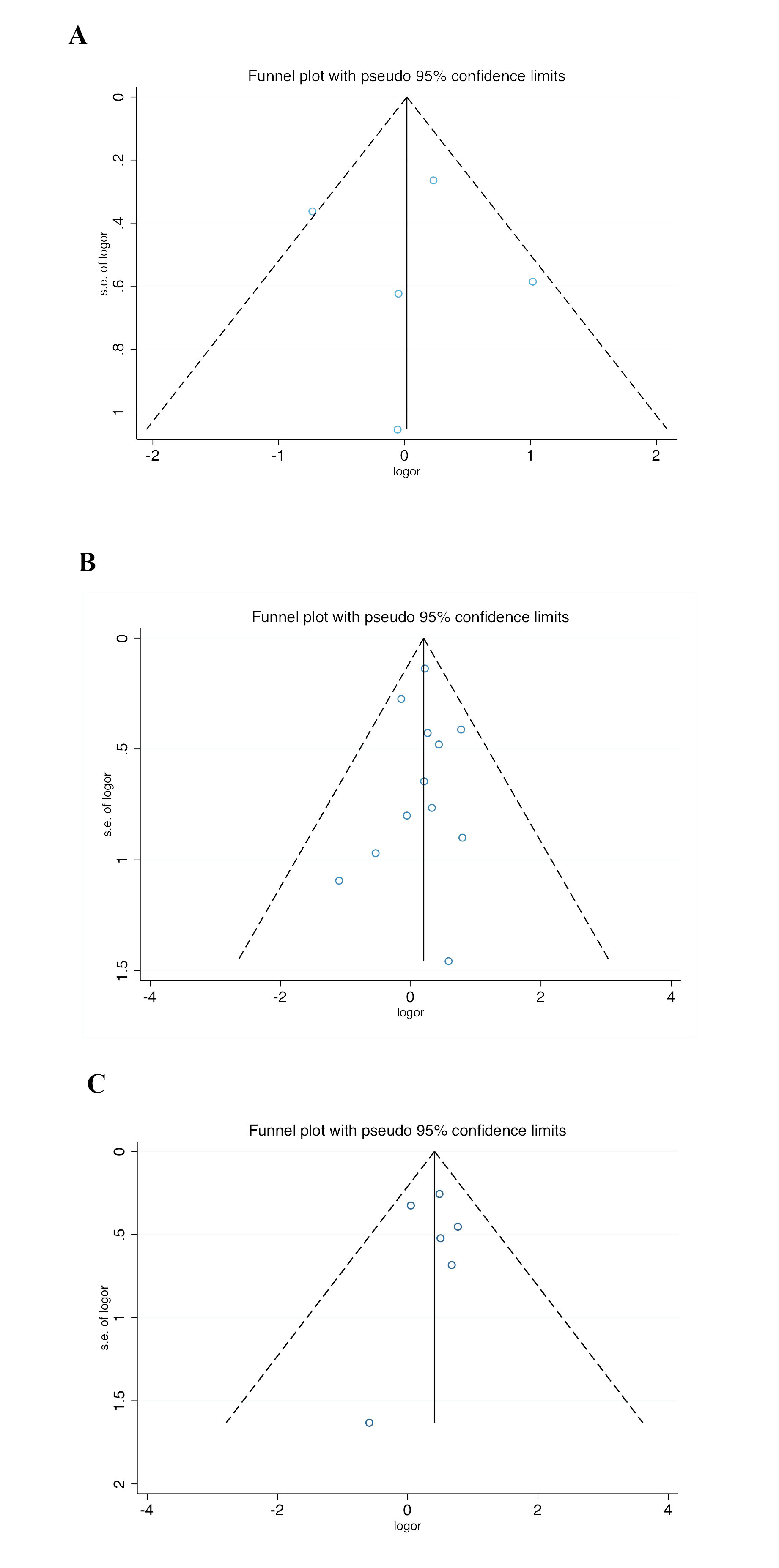
Supplementary Figures

**Supplementary Material 4** Funnel plots were performed to estimate publication bias. (**A**) infection. (**B**) mortality. (**C**) severity.

**
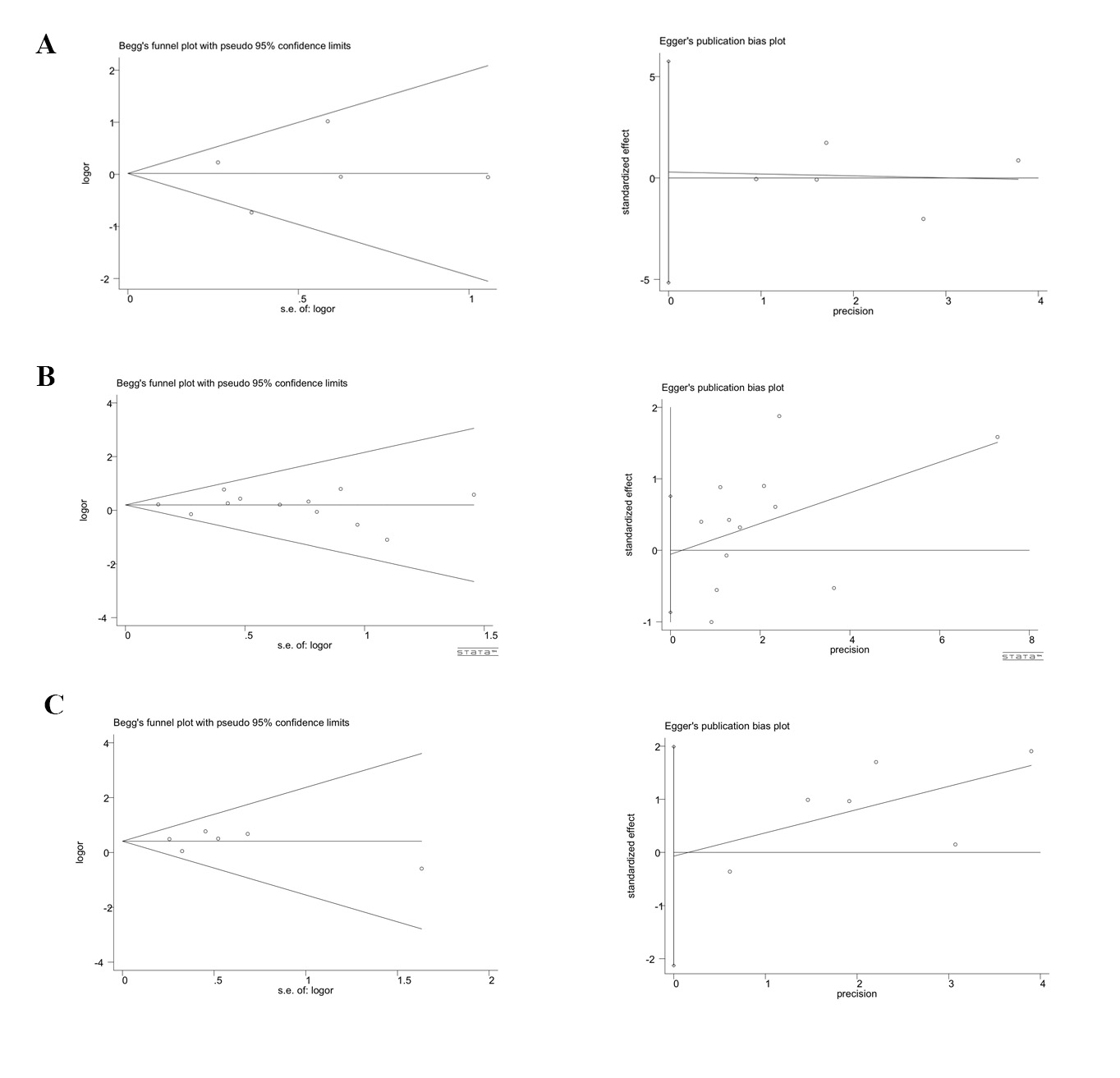
**

**Supplementary Material 5** Egger's tests and Begg's tests were performed to estimate publication bias after funnel plots. (**A**) infection. (**B**) mortality. (**C**) severity.

**
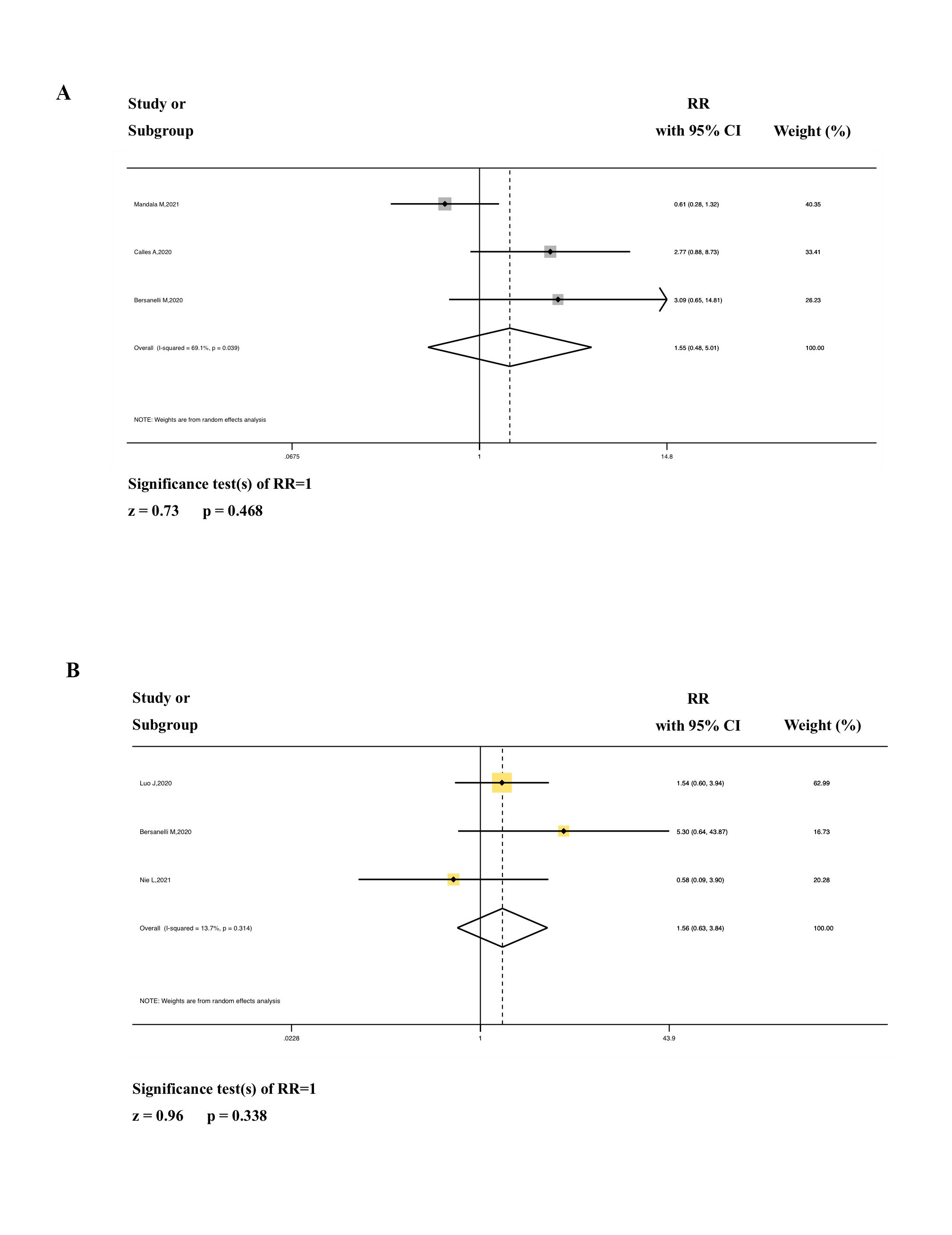
**

Supplementary Material 6 Forest plot of the analysis for the relationship between ICIs and SARS-CoV-2 infection in lung cancer patients. CI, confidence interval; RR, relative risk. (A) infection. (B) mortality.


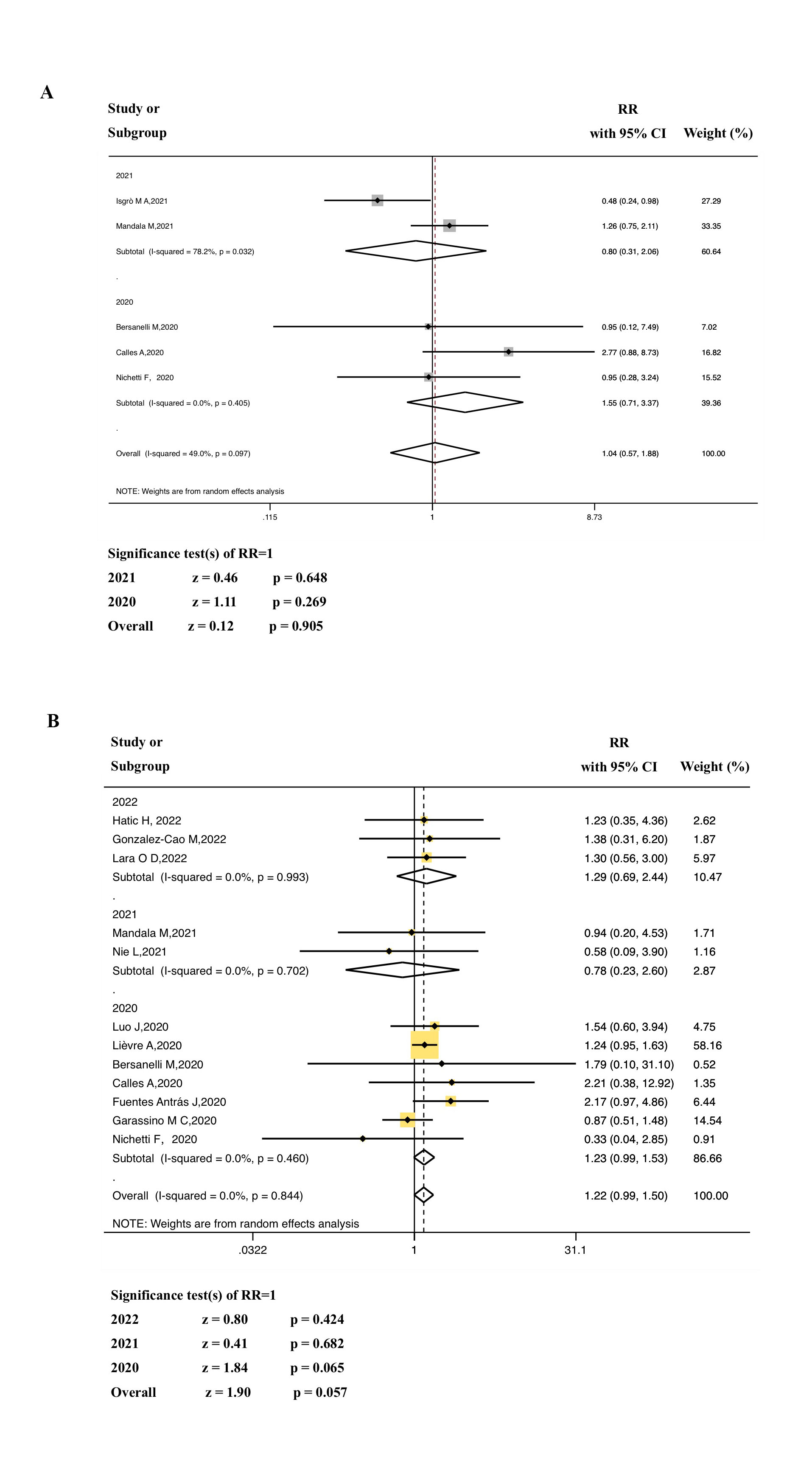


Supplementary Material 7 Forest plot of the Year subgroup analysis for the relationship between ICIs and SARS-CoV-2 infection. CI, confidence interval; RR, relative risk. (A) infection. (B) mortality.
